# Supplementary material for: Positive Mental Health Questionnaire (PMHQ) for Healthcare Workers: A Psychometric Evaluation
Source: Healthcare (Basel). 2023 Nov 26;11(23):3041. doi: 10.3390/healthcare11233041 (PMC10706672; doi:10.3390/healthcare11233041)
Supplement: Supplementary file 1 [file healthcare-11-03041-s001.zip › healthcare-2621324-supplementary.pdf]

healthcare

MDPI

Supplementary Materials

Table S1. A. Polychoric correlation matrix of the 39 items.

	PCDi	V1	V2	V3	V4	V5	V6	V7	V8	V9	V10	V11	V12	V13	V14	V15	V16	V17	V18	V19	V20	V21	V22	V23	V24	V25	V26	V27	V28	V29	V30	V31	V32	V33	V34	V35	V36	V37	V38	V39
V1	7.90%	-- --	0.339	0.056	-0.172	-0.086	0.416	-0.261	-0.278	-0.27	-0.231	0.053	0.281	0.222	0.223	0.182	0.193	0.092	0.098	0.318	0.364	-0.135	0.005	-0.142	0.246	0.248	0.308	0.314	0.267	0.165	0.244	-0.065	-0.305	-0.008	-0.192	-0.232	-0.169	-0.012	-0.006	0.034
V2	13.40%	0.339	-- --	-0.053	-0.185	-0.179	0.206	-0.204	0.028	0.038	-0.062	0.038	0.215	0.23	0.198	0.081	0.265	0.069	0.051	0.511	0.376	-0.239	-0.018	-0.205	0.431	0.254	-0.055	0.093	0.068	0.178	-0.012	0.035	-0.128	0.027	-0.058	-0.045	0.041	-0.09	-0.102	0.006
V3	52.30%	0.075	-0.071	-- --	0.281	0.255	0.085	0.11	0.283	0.355	0.286	0.135	-0.089	0.057	-0.066	-0.222	-0.256	0.023	-0.084	-0.105	0.061	0.157	0.471	0.174	-0.16	-0.088	0.063	-0.077	-0.129	-0.11	-0.103	0.217	0.208	0.272	0.428	0.417	0.232	0.326	0.232	0.347
V4	17.60%	-0.172	-0.185	0.376	-- --	0.409	-0.103	0.171	0.257	0.192	0.396	0.079	-0.098	-0.216	0.036	0.028	0.033	-0.052	0.047	-0.268	-0.24	0.271	0.361	0.158	-0.257	-0.065	0.121	0.017	0.127	0.09	-0.076	0.177	0.312	0.266	0.431	0.325	0.233	0.442	0.091	0.232
V5	51.40%	-0.115	-0.24	0.455	0.547	-- --	-0.108	0.112	0.146	0.172	0.281	0.123	-0.041	-0.101	-0.043	0.018	-0.034	-0.149	0.035	-0.226	-0.28	0.241	0.318	0.161	-0.355	-0.127	0.08	-0.096	0.039	-0.198	-0.02	0.189	0.309	0.267	0.353	0.342	0.237	0.375	0.169	0.311
V6	7.80%	0.416	0.206	0.114	-0.103	-0.144	-- --	-0.461	-0.362	-0.427	-0.436	-0.275	0.304	0.306	0.469	0.456	0.385	0.319	0.322	0.334	0.414	-0.049	-0.217	-0.167	0.197	0.326	0.374	0.422	0.359	0.299	0.47	-0.165	-0.383	0.05	-0.321	-0.266	-0.27	-0.27	-0.179	-0.17
V7	8.20%	-0.261	-0.204	0.146	0.171	0.149	-0.461	-- --	0.626	0.589	0.384	0.388	-0.214	-0.173	-0.266	-0.359	-0.197	-0.174	-0.219	-0.33	-0.345	0.237	0.256	0.113	-0.197	-0.129	-0.32	-0.349	-0.288	-0.283	-0.23	0.249	0.467	0.206	0.332	0.279	0.209	0.262	0.27	0.303
V8	10.80%	-0.278	0.028	0.378	0.257	0.196	-0.362	0.626	-- --	0.744	0.594	0.373	-0.318	-0.261	-0.347	-0.337	-0.251	-0.163	-0.204	-0.206	-0.251	0.196	0.361	0.358	-0.083	-0.068	-0.328	-0.317	-0.4	-0.149	-0.344	0.369	0.624	0.358	0.517	0.45	0.441	0.489	0.288	0.377
V9	10.60%	-0.27	0.038	0.475	0.192	0.23	-0.427	0.589	0.744	-- --	0.607	0.337	-0.278	-0.21	-0.258	-0.405	-0.241	-0.189	-0.246	-0.174	-0.216	0.246	0.423	0.22	-0.129	-0.104	-0.242	-0.284	-0.229	-0.311	-0.381	0.311	0.577	0.288	0.549	0.52	0.334	0.371	0.189	0.356
V10	10.20%	-0.231	-0.062	0.382	0.396	0.376	-0.436	0.384	0.594	0.607	-- --	0.234	-0.214	-0.109	-0.289	-0.376	-0.295	-0.227	-0.224	-0.226	-0.258	0.161	0.473	0.299	-0.032	-0.189	-0.283	-0.345	-0.293	-0.267	-0.434	0.358	0.586	0.304	0.649	0.549	0.437	0.529	0.292	0.416
V11	9.00%	0.053	0.038	0.18	0.079	0.164	-0.275	0.388	0.373	0.337	0.234	-- --	-0.149	-0.227	-0.54	-0.476	-0.278	-0.251	-0.445	-0.038	-0.333	0.108	0.337	0.25	-0.004	-0.213	-0.331	-0.309	-0.27	-0.329	-0.353	0.297	0.289	0.207	0.151	0.212	0.201	0.243	0.291	0.182
V12	48.10%	0.376	0.287	-0.16	-0.13	-0.074	0.406	-0.286	-0.425	-0.371	-0.286	-0.199	-- --	0.237	0.452	0.356	0.202	0.175	0.21	0.256	0.195	-0.112	-0.102	-0.082	0.154	0.133	0.14	0.189	0.205	0.085	0.152	-0.087	-0.238	-0.118	-0.201	-0.143	-0.131	-0.117	-0.06	-0.105

V13	47.30%	0.297	0.307	0.101	-0.288	-0.18	0.409	-0.231	-0.349	-0.28	-0.146	-0.304	0.423	-- --	0.453	0.221	0.199	0.201	0.223	0.288	0.294	-0.097	-0.04	-0.001	0.341	0.217	0.23	0.208	0.232	0.082	0.119	0.027	-0.212	-0.084	-0.125	-0.185	-0.198	-0.143	-0.079	0.019
V14	11.40%	0.223	0.198	-0.089	0.036	-0.058	0.469	-0.266	-0.347	-0.258	-0.289	-0.54	0.604	0.606	-- --	0.694	0.512	0.51	0.452	0.359	0.507	-0.077	-0.421	-0.312	0.122	0.379	0.464	0.374	0.476	0.386	0.478	-0.231	-0.209	-0.195	-0.244	-0.224	-0.365	-0.279	-0.28	-0.16
V15	9.50%	0.182	0.081	-0.296	0.028	0.024	0.456	-0.359	-0.337	-0.405	-0.376	-0.476	0.476	0.296	0.694	-- --	0.543	0.506	0.589	0.125	0.326	0.035	-0.514	-0.143	0.107	0.442	0.523	0.454	0.502	0.4	0.619	-0.265	-0.229	-0.163	-0.353	-0.334	-0.219	-0.382	-0.376	-0.305
V16	47.40%	0.258	0.354	-0.457	0.044	-0.061	0.514	-0.263	-0.336	-0.322	-0.394	-0.371	0.361	0.355	0.685	0.726	-- --	0.494	0.434	0.061	0.141	0.077	-0.392	-0.216	0.003	0.475	0.28	0.209	0.272	0.254	0.375	-0.174	-0.226	-0.178	-0.329	-0.337	-0.204	-0.197	-0.357	-0.214
V17	11.30%	0.092	0.069	0.031	-0.052	-0.2	0.319	-0.174	-0.163	-0.189	-0.227	-0.251	0.234	0.269	0.51	0.506	0.66	-- --	0.658	0.04	0.215	0.032	-0.282	-0.185	0.096	0.292	0.233	0.202	0.222	0.273	0.248	-0.029	-0.217	-0.179	-0.176	-0.094	-0.114	-0.24	-0.248	-0.143
V18	8.80%	0.098	0.051	-0.112	0.047	0.047	0.322	-0.219	-0.204	-0.246	-0.224	-0.445	0.28	0.298	0.452	0.589	0.58	0.658	-- --	0.09	0.192	0.068	-0.222	-0.175	0.053	0.342	0.312	0.359	0.267	0.321	0.397	-0.091	-0.177	-0.126	-0.054	-0.248	-0.18	-0.168	-0.362	-0.205
V19	9.80%	0.318	0.511	-0.141	-0.268	-0.303	0.334	-0.33	-0.206	-0.174	-0.226	-0.038	0.342	0.384	0.359	0.125	0.082	0.04	0.09	-- --	0.502	-0.14	-0.154	-0.245	0.421	0.249	0.129	0.17	0.154	0.224	0.146	-0.04	-0.245	-0.093	-0.119	-0.145	-0.037	-0.218	-0.016	-0.208
V20	6.50%	0.364	0.376	0.081	-0.24	-0.374	0.414	-0.345	-0.251	-0.216	-0.258	-0.333	0.26	0.393	0.507	0.326	0.189	0.215	0.192	0.502	-- --	-0.168	-0.12	-0.462	0.415	0.44	0.322	0.202	0.26	0.228	0.348	0.05	-0.298	-0.117	-0.047	-0.214	-0.18	-0.149	-0.061	0.016
V21	51.70%	-0.18	-0.32	0.28	0.362	0.431	-0.065	0.317	0.262	0.329	0.215	0.145	-0.199	-0.173	-0.103	0.047	0.137	0.043	0.091	-0.187	-0.224	-- --	0.23	0.16	-0.249	0	0.027	-0.014	0.002	-0.109	0.003	0.171	0.227	0.217	0.261	0.253	0.18	0.248	0.084	0.162
V22	14.40%	0.005	-0.018	0.63	0.361	0.425	-0.217	0.256	0.361	0.423	0.473	0.337	-0.137	-0.053	-0.421	-0.514	-0.524	-0.282	-0.222	-0.154	-0.12	0.307	-- --	0.36	-0.06	-0.193	-0.239	-0.291	-0.311	-0.252	-0.3	0.342	0.4	0.361	0.549	0.489	0.467	0.588	0.28	0.376
V23	9.50%	-0.142	-0.205	0.232	0.158	0.215	-0.167	0.113	0.358	0.22	0.299	0.25	-0.11	-0.001	-0.312	-0.143	-0.289	-0.185	-0.175	-0.245	-0.462	0.213	0.36	-- --	-0.058	-0.24	-0.102	-0.061	-0.248	0.017	-0.225	0.15	0.39	0.227	0.179	0.257	0.276	0.292	0.133	0.217
V24	16.30%	0.246	0.431	-0.213	-0.257	-0.474	0.197	-0.197	-0.083	-0.129	-0.032	-0.004	0.206	0.456	0.122	0.107	0.004	0.096	0.053	0.421	0.415	-0.333	-0.06	-0.058	-- --	0.306	0.051	0.084	0.073	0.27	-0.006	0.015	-0.111	-0.069	-0.155	-0.154	-0.055	-0.184	0.006	-0.032
V25	11.40%	0.248	0.254	-0.117	-0.065	-0.17	0.326	-0.129	-0.068	-0.104	-0.189	-0.213	0.178	0.29	0.379	0.442	0.635	0.292	0.342	0.249	0.44	0	-0.193	-0.24	0.306	-- --	0.245	0.227	0.328	0.309	0.368	-0.167	-0.089	0.042	-0.087	-0.309	-0.095	-0.058	-0.182	-0.186
V26	5.40%	0.308	-0.055	0.084	0.121	0.107	0.374	-0.32	-0.328	-0.242	-0.283	-0.331	0.188	0.308	0.464	0.523	0.375	0.233	0.312	0.129	0.322	0.036	-0.239	-0.102	0.051	0.245	-- --	0.558	0.722	0.286	0.435	-0.154	-0.205	0.06	-0.175	-0.21	-0.229	-0.11	-0.327	-0.206
V27	4.70%	0.314	0.093	-0.103	0.017	-0.128	0.422	-0.349	-0.317	-0.284	-0.345	-0.309	0.252	0.278	0.374	0.454	0.279	0.202	0.359	0.17	0.202	-0.019	-0.291	-0.061	0.084	0.227	0.558	-- --	0.532	0.374	0.458	-0.179	-0.22	0.036	-0.208	-0.22	-0.203	-0.174	-0.39	-0.132

V28	6.00%	0.267	0.068	-0.172	0.127	0.052	0.359	-0.288	-0.4	-0.229	-0.293	-0.27	0.274	0.31	0.476	0.502	0.363	0.222	0.267	0.154	0.26	0.002	-0.311	-0.248	0.073	0.328	0.722	0.532	-- --	0.241	0.367	-0.161	-0.234	0.058	-0.255	-0.337	-0.397	-0.249	-0.293	-0.25
V29	8.60%	0.165	0.178	-0.148	0.09	-0.265	0.299	-0.283	-0.149	-0.311	-0.267	-0.329	0.113	0.11	0.386	0.4	0.339	0.273	0.321	0.224	0.228	-0.145	-0.252	0.017	0.27	0.309	0.286	0.374	0.241	-- --	0.419	-0.259	-0.209	-0.08	-0.285	-0.333	-0.2	-0.21	-0.246	-0.289
V30	6.10%	0.244	-0.012	-0.138	-0.076	-0.027	0.47	-0.23	-0.344	-0.381	-0.434	-0.353	0.204	0.16	0.478	0.619	0.501	0.248	0.397	0.146	0.348	0.004	-0.3	-0.225	-0.006	0.368	0.435	0.458	0.367	0.419	-- --	-0.239	-0.249	0.031	-0.34	-0.265	-0.265	-0.275	-0.286	-0.231
V31	49.80%	-0.087	0.047	0.387	0.236	0.337	-0.221	0.332	0.494	0.415	0.478	0.396	-0.156	0.048	-0.309	-0.354	-0.311	-0.038	-0.121	-0.054	0.066	0.305	0.458	0.201	0.02	-0.223	-0.206	-0.24	-0.215	-0.347	-0.32	-- --	0.3	0.212	0.295	0.284	0.228	0.241	0.144	0.245
V32	12.40%	-0.305	-0.128	0.278	0.312	0.413	-0.383	0.467	0.624	0.577	0.586	0.289	-0.318	-0.283	-0.209	-0.229	-0.302	-0.217	-0.177	-0.245	-0.298	0.303	0.4	0.39	-0.111	-0.089	-0.205	-0.22	-0.234	-0.209	-0.249	0.4	-- --	0.49	0.489	0.521	0.457	0.52	0.175	0.358
V33	50.90%	-0.011	0.036	0.485	0.356	0.476	0.066	0.275	0.479	0.385	0.406	0.277	-0.21	-0.15	-0.261	-0.217	-0.319	-0.239	-0.168	-0.125	-0.157	0.387	0.482	0.304	-0.093	0.056	0.08	0.048	0.078	-0.106	0.042	0.379	0.654	-- --	0.382	0.335	0.359	0.409	0.104	0.241
V34	14.30%	-0.192	-0.058	0.571	0.431	0.471	-0.321	0.332	0.517	0.549	0.649	0.151	-0.268	-0.167	-0.244	-0.353	-0.44	-0.176	-0.054	-0.119	-0.047	0.349	0.549	0.179	-0.155	-0.087	-0.175	-0.208	-0.255	-0.285	-0.34	0.394	0.489	0.511	-- --	0.649	0.503	0.557	0.288	0.476
V35	13.70%	-0.232	-0.045	0.558	0.325	0.457	-0.266	0.279	0.45	0.52	0.549	0.212	-0.191	-0.247	-0.224	-0.334	-0.451	-0.094	-0.248	-0.145	-0.214	0.337	0.489	0.257	-0.154	-0.309	-0.21	-0.22	-0.337	-0.333	-0.265	0.379	0.521	0.448	0.649	-- --	0.515	0.438	0.294	0.4
V36	11.30%	-0.169	0.041	0.31	0.233	0.316	-0.27	0.209	0.441	0.334	0.437	0.201	-0.175	-0.265	-0.365	-0.219	-0.272	-0.114	-0.18	-0.037	-0.18	0.24	0.467	0.276	-0.055	-0.095	-0.229	-0.203	-0.397	-0.2	-0.265	0.304	0.457	0.48	0.503	0.515	-- --	0.526	0.164	0.318
V37	13.50%	-0.012	-0.09	0.435	0.442	0.502	-0.27	0.262	0.489	0.371	0.529	0.243	-0.156	-0.191	-0.279	-0.382	-0.263	-0.24	-0.168	-0.218	-0.149	0.331	0.588	0.292	-0.184	-0.058	-0.11	-0.174	-0.249	-0.21	-0.275	0.322	0.52	0.547	0.557	0.438	0.526	-- --	0.22	0.439
V38	11.70%	-0.006	-0.102	0.31	0.091	0.226	-0.179	0.27	0.288	0.189	0.292	0.291	-0.08	-0.106	-0.28	-0.376	-0.477	-0.248	-0.362	-0.016	-0.061	0.112	0.28	0.133	0.006	-0.182	-0.327	-0.39	-0.293	-0.246	-0.286	0.193	0.175	0.139	0.288	0.294	0.164	0.22	-- --	0.301
V39	50.10%	0.045	0.008	0.62	0.31	0.555	-0.228	0.405	0.504	0.476	0.556	0.243	-0.187	0.033	-0.214	-0.408	-0.382	-0.192	-0.274	-0.278	0.022	0.29	0.503	0.29	-0.043	-0.248	-0.276	-0.177	-0.334	-0.387	-0.309	0.437	0.479	0.43	0.636	0.534	0.424	0.586	0.402	-- --

Percentage of covariance destroyed in each variable.

Table S1. B. Factorial loads per factor for each model.

	1 factor model	4 factor model				6 factors model					
Item	F1	F1	F2	F3	F4	F1	F2	F3	F4	F5	F6
R4	0.47	0.59				0.60					
R6	0.45	0.56				0.56					
R7	0.44	0.55				0.55					
R12	0.66	0.81				0.82					
R14	0.68	0.84				0.84					
R31	0.65	0.81				0.81					
R38	0.42	0.56				0.56					
R39	0.47	0.62				0.63					
R1	0.36		0.43				0.17				
R3	0.23		0.33				0.35				
R23	0.55		0.67				0.76				
R25	0.38		0.50				0.59				
R37	0.50		0.64				0.75				
R8	0.38		0.49					0.53			
R9	0.47		0.59					0.66			
R11	0.34		0.45					0.83			
R18	0.64		0.78					0.80			
R20	0.39		0.48					0.79			
R24	0.29		0.40						0.73		
R30	0.39		0.46						0.66		
R10	0.54			0.73					0.76		
R13	0.48			0.66					0.55		
R19	0.56			0.75					0.72		
R33	0.43			0.55						0.12	
R34	0.54			0.72						0.77	
R15	0.51				0.53					0.63	
R16	0.68				0.72					0.79	
R17	0.56				0.60					0.74	
R27	0.70				0.74					0.61	
R28	0.66				0.70					0.71	
R29	0.54				0.57					0.42	
R32	0.62				0.66					0.75	
R35	0.39				0.40						0.40
R36	0.66				0.70						0.61
R2	0.55				0.57						0.46
R5	0.59				0.62						0.81
R21	0.76				0.79						0.50
R22	0.73				0.76						0.41
R26	0.71				0.74						0.49

F: Factor

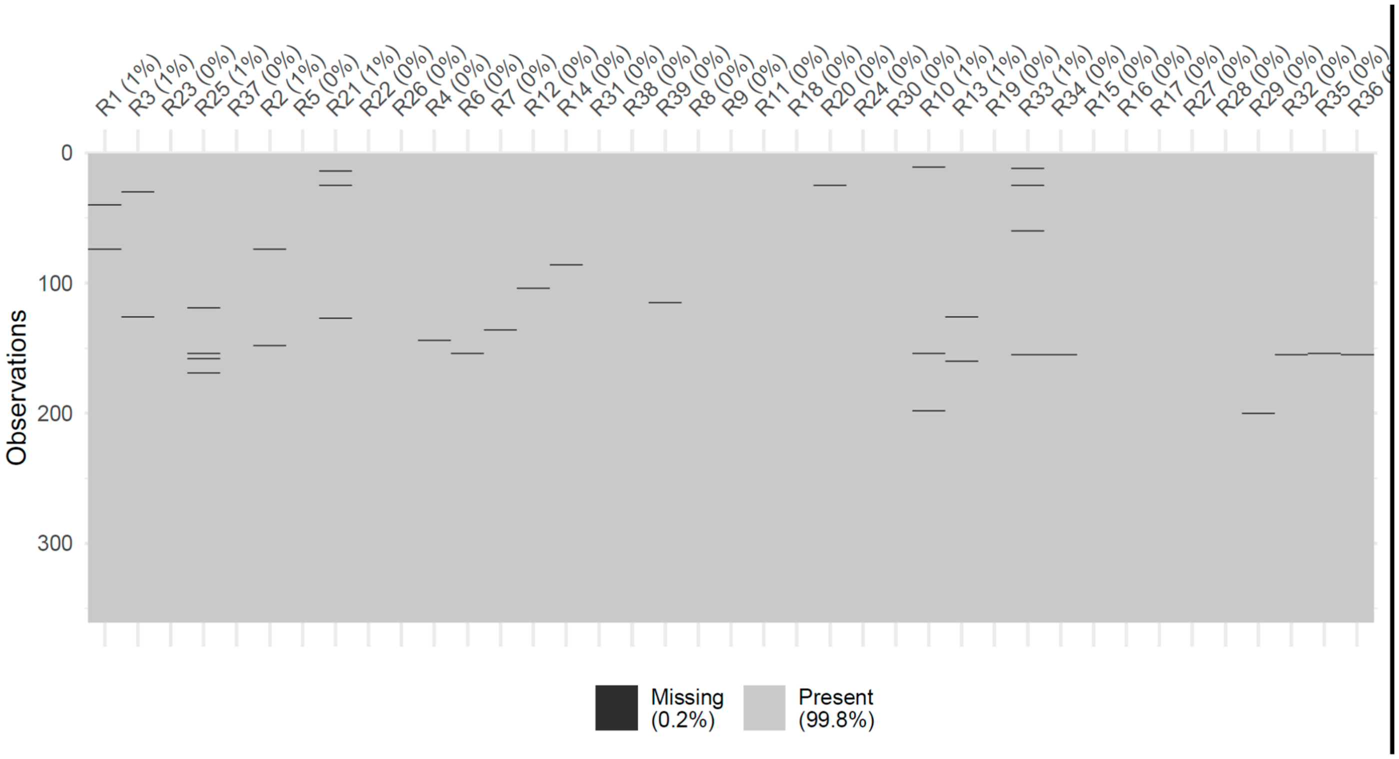

Figure S1. A. Missing data pattern for Positive Mental Health Questionnaire (PMHQ).
